# Percutaneous recanalization of non-cirrhotic extrahepatic portal vein obstruction in children: technical considerations in a preliminary cohort

**DOI:** 10.1007/s00330-024-11040-8

**Published:** 2024-09-06

**Authors:** Paolo Marra, Stephanie Franchi-Abella, José A. Hernandez, Maxime Ronot, Riccardo Muglia, Lorenzo D’Antiga, Sandro Sironi

**Affiliations:** 1 Department of Radiology, ASST Papa Giovanni XXIII Hospital, School of Medicine and Surgery, University of Milano Bicocca, Bergamo, Italy; 2 Department of Pediatric Radiology, DMU Smart Imaging, Bicêtre Hospital, AP-HP. Reference Centre for Vascular Diseases of the Liver, FSMR FILFOIE, ERN RARE LIVER, FHU Hépatinov, Le Kremlin-Bicêtre, France; 3 BIOMAPS UMR 9011 CNRS—INSERM—CEA, Paris-Saclay University, Paris, France; 4 Department of Interventional Radiology, Texas Children’s Hospital, Baylor College of Medicine, Houston, TX USA; 5 Department of Radiology, Baylor College of Medicine, Houston, TX USA; 6 Service de Radiologie, Hôpital Beaujon APHP Nord, Clichy & Université Paris Cité, CRI, INSERM, Paris, France; 7 Pediatric Hepatology, Gastroenterology and Transplantation, ASST Papa Giovanni XXIII Hospital, Bergamo, Italy; 8 Department of Medicine and Surgery, University of Milano Bicocca, Milan, Italy

**Keywords:** Portal hypertension, Extrahepatic portal vein obstruction, Portal vein thrombosis, Meso-Rex bypass, Portal vein recanalization

## Abstract

**Objectives:**

Portal hypertension resulting from non-cirrhotic extrahepatic portal vein obstruction (EHPVO) in children has been primarily managed with the Meso-Rex bypass, but only a few patients have a viable Rex recessus, required by surgery. This study reports a preliminary series of patients who underwent interventional radiology attempts at portal vein recanalization (PVR), with a focus on technical aspects and safety.

**Methods:**

A retrospective review of consecutive patients with severe portal hypertension due to non-cirrhotic EHPVO at a single institution from 2022, who underwent percutaneous attempts at PVR, was performed. Technical and clinical data including fluoroscopy time, radiation exposure, technical and clinical success, complications and follow-up were recorded.

**Results:**

Eleven patients (6 males and 5 females; median age 7 years, range 1–14) underwent 15 percutaneous transhepatic (*n* = 1), transplenic (*n* = 11), or simultaneous transhepatic/transplenic (*n* = 3) procedures. Rex recessus was patent in 4/11 (36%). Fluoroscopy resulted in a high median total dose area product (DAP) of 123 Gycm^2^ (range 17–788 Gycm^2^) per procedure. PVR was achieved in 5/11 patients (45%), 3/5 with obliterated Rex recessus. Two adverse events of grade 2 and grade 3 occurred without sequelae. After angioplasty, 4/5 patients required stenting to obtain sustained patency, as demonstrated by colour-Doppler ultrasound in all PVR after a median follow-up of 6 months (range 6–14).

**Conclusion:**

Our preliminary experience suggests that 45% of children with non-cirrhotic EHPVO can restore portal flow even with obliterated Rex recessus. In non-cirrhotic EHPVO, PVR may be an option, if a Meso-Rex bypass is not feasible, although the radiation exposure deserves attention.

**Clinical relevance statement:**

Innovative percutaneous procedures may have the potential to be an alternative option to the traditional surgical approach in the management of non-cirrhotic EHPVO and its complications in children not eligible for Meso-Rex bypass surgery.

**Key Points:**

- *Non-cirrhotic portal hypertension in children has been traditionally managed by surgery with Meso-Rex bypass creation.*
- *Percutaneous PVR may restore the patency of the native portal system even when the Rex recessus is obliterated and surgery has been excluded.*
- *Interventional radiological techniques may offer a minimally invasive solution in complex cases of EHPVO in children when Meso-Rex bypass is not feasible.*

## Introduction

Non-cirrhotic extrahepatic portal vein obstruction (EHPVO) resulting from portal vein thrombosis is a primary cause of portal hypertension in children. It typically presents with splenomegaly and gastrointestinal bleeding, often leading to life-threatening situations [1]. A comprehensive multicentre national study [2] conducted in Italy, involving 187 children, identified prematurity, a history of umbilical vein catheterization and neonatal illnesses as prevalent factors associated with this pathological condition. While medical therapy and endoscopic procedures can achieve control over portal hypertension, the study found that up to 34% of children required surgery or transjugular intrahepatic portosystemic shunt (TIPS) creation during an 11.3-year follow-up. Historically, and according to the Baveno VII consensus [3, 4], surgical Meso-Rex bypass is recommended as the standard of care (level 2 of evidence, grade B recommendation) for all children with complications of portal cavernoma. However, in order to perform the Meso-Rex bypass, it is essential to confirm the patency of the Rex recessus, a prerequisite reported to be present in only half of the cases [5]. Moreover, surgery may be burdened by a high intraoperative failure rate and postoperative complications, mainly involving bypass thrombosis [6, 7]. Percutaneous procedures are considered secondary and are typically reserved for refractory conditions (level 2 of evidence; grade C recommendation). There are compelling arguments supporting the prioritization of percutaneous approaches, which now extend far beyond traditional TIPS procedures. These arguments encompass the adoption of new, innovative percutaneous techniques already adopted in adults [8] and herein illustrated, which pediatric interventional radiologists should become thoroughly familiar with. The aim of this study is to report an illustrative case series of patients who underwent percutaneous attempts at portal vein recanalization (PVR) prior to considering any type of other intervention, with a focus on technical success and safety, to increase awareness of these evolving treatments options among pediatric multidisciplinary boards.

## Material and methods

This retrospective study presents a case series involving 11 consecutive patients who underwent percutaneous attempts at PVR following multidisciplinary evaluation between January 2022 and February 2024 at a single centre with 20 years of experience in both Meso-Rex bypass surgery and pediatric interventional treatments, including complex percutaneous hepatobiliary interventions like percutaneous portal vein catheterization, embolization, angioplasty, stenting, and TIPS. All patients exhibited non-cirrhotic portal hypertension suspected to stem from EHPVO resulting from acquired chronic portal vein thrombosis during post-natal age. Consent was obtained from all patients (or their legal representatives) involved in the study which was approved by the Institutional Review Board. Discussions surrounding these cases involved an international team of experts in liver diagnostic and interventional radiology.

### Preoperative workup

For every pediatric patient presenting with suspected EHPVO the diagnostic work-up in our centre included a complete clinical assessment, a panel of liver function laboratory tests, an upper gastrointestinal endoscopy and abdominal colour-Doppler ultrasound and CT angiography. If common causes of acquired portal vein thrombosis (i.e. perinatal sicknesses) were ruled out a screening for coagulation disorders was performed. Furthermore, if no clinical signs of chronic liver disease emerged and a colour-Doppler ultrasound of the liver did not reveal any obvious native (i.e. non-cavernous) intrahepatic portal vessel, the patient was screened for Rex recessus patency through transjugular wedge hepatic venography. Afterwards, regardless of the status of Rex recessus patency, which is only required for Meso-Rex bypass surgery, the patient was listed for an attempt at PVR by means of a percutaneous approach.

### PVR technique

**Fig. 1:**
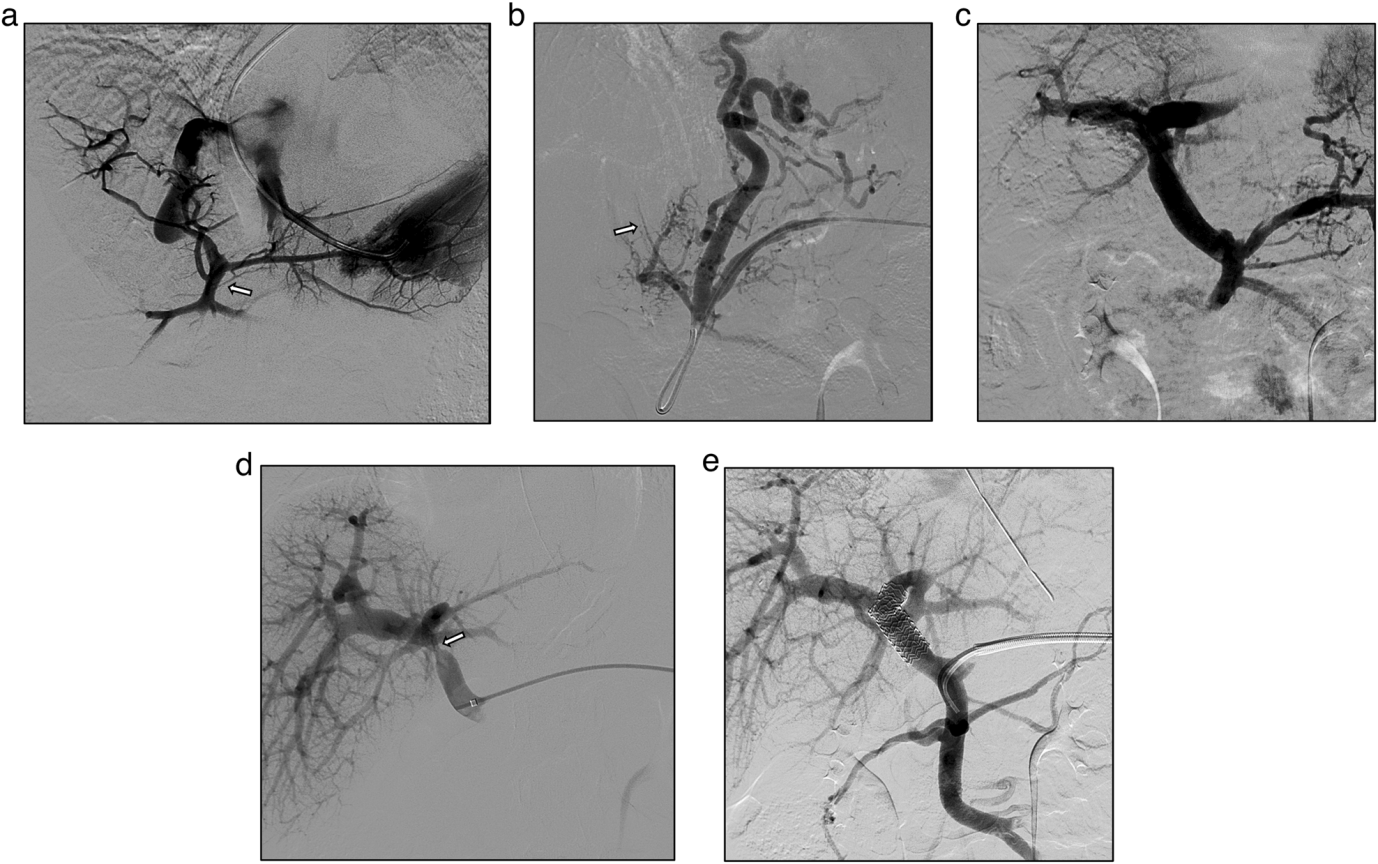
A 7-year-old female with a history of perinatal umbilical vein catheterization developed severe hematemesis. **a** shows transjugular wedge hepatic venography with patent Rex recessus (arrow). A percutaneous transplenic venography was performed (**b**); the arrow indicates the relic of the thrombosed portal vein, which was successfully recanalized and dilated, as shown in **c**. After the procedure, platelet counts normalized. **d** shows a recurrent stricture (arrow) of the distal extrahepatic portal vein just before the bifurcation that was treated with angioplasty and stenting (**e**). Portal vein patency was demonstrated after a 10-month follow-up and no further episodes of gastrointestinal bleeding occurred

**Fig. 2:**
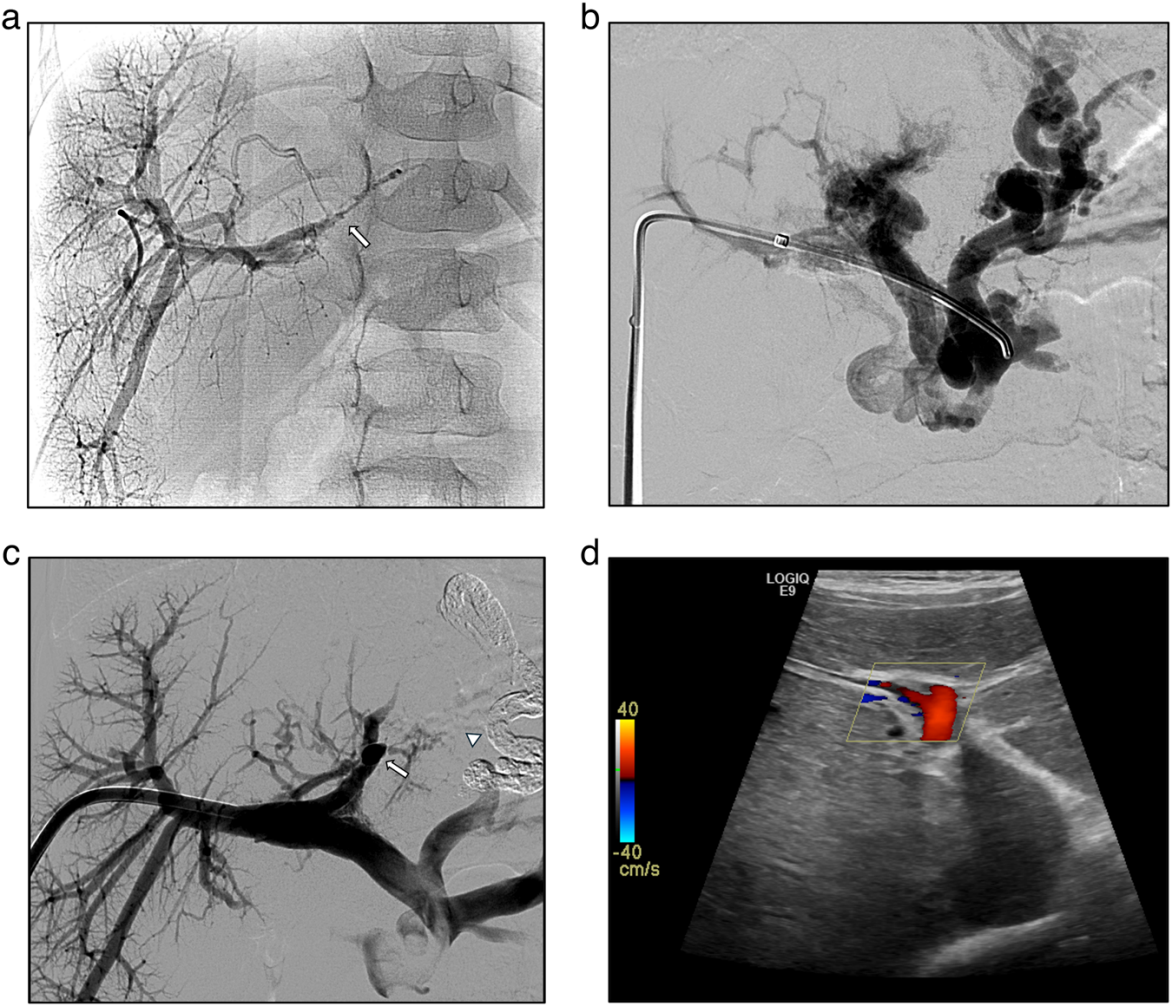
A 4-year-old male with a history of perinatal umbilical vein catheterization presented with severe hematemesis. **a** shows percutaneous transhepatic portal venography with opacification of right intrahepatic portal branches and partial thrombosis of the Rex recessus (arrow). A transhepatic retrograde recanalization of the extrahepatic portal tract was performed. **b** shows spleno-mesenteric venography with cavernous transformation and left gastric varices. **c** shows the successful complete recanalization of the portal system, including the Rex recessus, and variceal glue embolization (arrowhead). Gastric varices disappeared at the endoscopic examination performed after 3 months. Platelet count normalization and portal vein patency at colour-Doppler ultrasound (**d**) were maintained during the 6-month follow-up

**Fig. 3:**
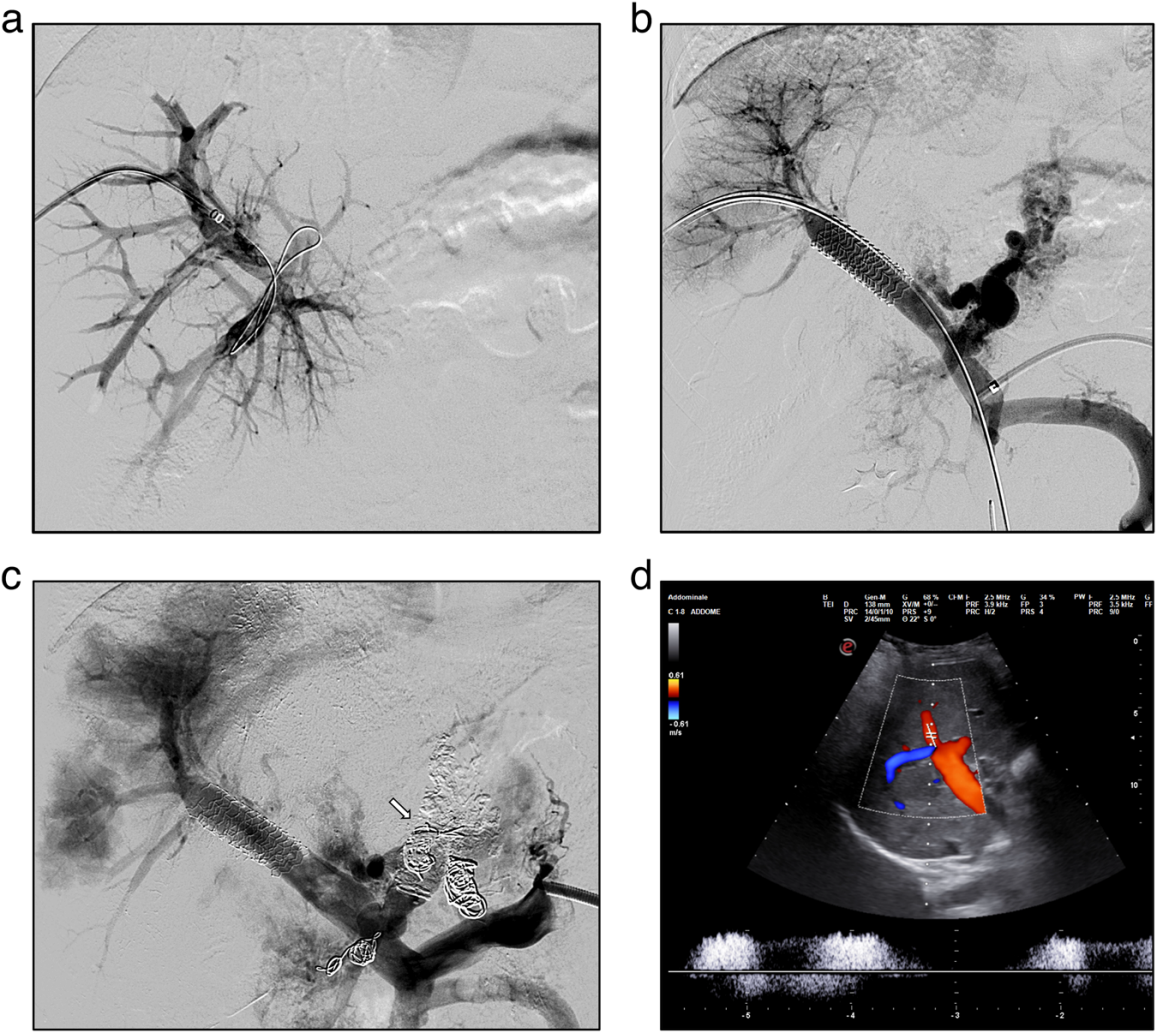
A 10-year-old male with a history of portal hypertension secondary to chronic portal vein thrombosis was unfit for Meso-Rex surgery due to Rex recessus thrombosis. **a** shows percutaneous transhepatic portal venography with partial opacification of the right intrahepatic portal branches and thrombosis of the remaining portal system. After successful recanalization by a combined retrograde transhepatic and antegrade transplenic approach with angioplasty and stenting. **b** shows opacification of part of the right liver sector with persistent gastric varices, which were subsequently embolized with coils and glue to reduce the steal phenomenon (arrow, **c**). Colour-Doppler ultrasound shows patency of the right branches of the portal vein with hepatopetal flow at a 4-month follow-up (**d**). After the procedure, the platelet counts normalized

All the procedures were performed under general anesthesia by dedicated pediatric interventional radiologists with at least five years of experience or training, in an angiographic suite equipped with a single-plane system (Allura Xper FD20, Philips Healthcare). The anesthesiologists came from a dedicated pediatric intensive care unit where they were specifically trained in cardiothoracic surgery and hepatobiliary interventions. The choice between the transhepatic, transplenic, or both simultaneous routes, was based on the visualization of native intrahepatic portal vessels with colour-Doppler ultrasound. If detectable, a right segmental branch of the portal vein was targeted under ultrasound guidance and cannulated under fluoroscopic guidance using a Neff introducer set (Cook Medical). Access to the portal vein was preferred through right caudal segments. If no native intrahepatic portal branches were seen, the splenic vein was catheterized through the inferior third of the spleen parenchyma as previously reported [9], with the same percutaneous introducer set. Owing to the unlikely visualization of intrahepatic portal vessels in most cases, anterograde transplenic access was usually performed. A 5 F vascular catheter (Berenstein, Cobra, Simmons, SOS; Cordis, Terumo, Soft Vu) was inserted through the introducer sheath and navigated up to the site of portal vein obstruction. To ensure the stability of the transplenic access, the Neff introducer was exchanged with a 24-cm 6 F introducer sheath (Super Arrow-Flex®, Teleflex). Recanalization was first attempted using a 0.035-inch angulated hydrophilic guidewire (Terumo) under fluoroscopy guidance (Fig. 1). For tight obstructions, recanalization of the thin vestige of the portal vein identified on venography was attempted with 1.8/1.9 F microcatheters (Carnelian, Tokai; ProGreat Lambda, Terumo) and hydrophilic 0.014” guidewires (Transend, Boston Scientific; Command, Abbott). Sharp recanalization was never performed. The retrograde transhepatic approach, although anatomically favourable, was feasible in a minority of cases (Fig. 2). Both the transhepatic and the transplenic accesses were necessary (Fig. 3) when PVR was not achievable through single access, providing a target for the fluoroscopy-guided recanalization. If PVR succeeded, angioplasty was performed with over-the-wire non-compliant balloon catheters and microcatheters of increasing size, starting from 6 mm up to over 30% of the estimated target vessel calibre (Mustang 0.035”, Sterling 0.018”, Boston Scientific). Balloon length ranged between 4 cm and 6 cm. After angioplasty, a portogram was obtained below the obstruction. Primary or secondary stenting were respectively considered in cases of balloon catheter recoil and residual stenosis, or when the obstruction recurred. Stenting was always performed with bare-metal devices through the transplenic access. Stent length was chosen in order to precisely cover the obstruction tract, avoiding the spleno-mesenteric confluence, If the obstruction involved the portal bifurcation, stent landing was allowed in the intrahepatic branches and cross-mesh dilation was performed to provide flow to collateral vessels through the mesh. In infants, 8–10-mm × 19–29-mm balloon-expandable Cobalt Chromium stents (Omnilink Elite, Abbott) were preferred, to allow post-dilation with patient growth. In adolescents, 12-mm × 40-mm self-expandable stainless-steel stents (WallStent, Boston Scientific) were employed. For Wallstent placement, the 6 F introducer sheath was exchanged with a 12-cm 9 F vascular introducer sheath (Ultimum, Abbott). The portocaval gradient was not measured. Variceal embolization with coils and/or glue was performed whenever spleno-mesenteric venography showed a flow steal phenomenon. Unfractionated heparin was routinely administered after the first angioplasty at a dose of 50 international units per kilogram. In cases of failed recanalization, the procedure was usually abandoned after 180 min of fluoroscopy and a second delayed attempt might be considered. Hemostasis of the percutaneous access was obtained through parenchymal tract embolization with glue, as previously described [9].

### Postoperative management

Every patient with successful recanalization underwent a strict follow-up with colour-Doppler ultrasound 24 h, 72 h, 1 week, 2 weeks, 1 month, 3 months, 6 months, and 1 year after the procedure, then yearly. Subcutaneous low molecular weight heparin at a prophylactic dosage (20 mg/d, reduced to 10 mg/d for children < 25 kgs) was maintained for at least 3 months in all cases while the therapeutic dosage (1 mg/kg/bid) was prescribed in cases of acute thrombosis detected intraoperatively or during postprocedural imaging surveillance. Antiplatelet therapy was exceptionally administered. If complete thrombosis of the portal vein or restenosis were detected during the follow-up, a new percutaneous procedure was considered.

### Outcome measures

Technical aspects of the procedures were collected and reviewed, including: approach, number of procedures, fluoroscopy and overall procedure time, dose area product (DAP), complications, technical success in terms of partial or complete restoration of the native portal vein, portal vein patency during follow-up, and clinical success in terms of portal hypertension control (endoscopic assessment of varices when available; platelet count; GI bleeding episodes). Oesophageal and gastric varices were graded according to Garcia-Tsao et al [10]. Complications were graded according to the CIRSE classification system [11].

## Results

Demographic and baseline clinical data of the study population and procedural data are synthesized in Tables 1 and 2, respectively. Individual baseline clinical data and procedural data are listed in Table S1. Briefly, among 11 patients (6 males and 5 females; median age 7 years; range 1–14), Rex recessus was patent in 4/11 (36%) while obstruction of the spleno-mesenteric confluence was observed in 2/11 (18%). Most of the patients had a history of umbilical vein catheterization and all presented hypersplenism. High-risk varices and a history of gastrointestinal bleeding were observed in 4 and 7 patients, respectively. A total of 15 percutaneous transhepatic (*n* = 1), transplenic (*n* = 11), or simultaneous transhepatic/transplenic (*n* = 3) procedures were performed with successful recanalization achieved in 5/11 patients (45%), 3/5 with obliterated Rex recessus. Outcomes data of successful PVR are synthesized in Table 3. Individual outcomes and procedure technical details are listed in Table S2. After successful angioplasty, 4/5 patients required metal stenting. A second procedure was required in four patients: three underwent secondary stenting due to restenosis at the distal part of the main portal trunk; one underwent primary stenting and required a second procedure due to acute postoperative stent thrombosis. Two patients had adverse events of grade 3 and grade 2, respectively: one intrahepatic arterial pseudoaneurysm that was effectively treated by transcatheter embolization and one hemoperitoneum that was medically managed. Ionizing radiation exposure data is summarized in Table 2 and detailed in Table S1. A median fluoroscopy time of 114 min (range 18–178 min), a median overall procedure time of 220 min (range 67–358 min) and a median total DAP of 123 Gycm^2^ (range 17–788 Gycm^2^) per procedure were recorded. All the patients with successful recanalization were in good clinical condition and presented sustained patency of the portal vein on colour-Doppler ultrasound imaging at a median follow-up of 6 months (range 6–14). The longest follow-up with the patency of the portal vein was 14 months. Clinical improvement of portal hypertension was demonstrated by a significant (*p* < 0.05) increase in platelet counts from a median of 89 × 109/L (range 72–174 × 109/L) to a median of 170 × 109/L (range 148–266 × 109/L); by the absence of further episodes of gastrointestinal bleeding and by a general improvement of the appearance of varices at endoscopy, when performed (see Tables S1 and S2). Four of six patients who failed PVR received TIPS (*n* = 2) landing on cavernous vessels, splenectomy (*n* = 1), or surgical meso-renal shunt (*n* = 1) due to the non-feasibility of the Meso-Rex bypass, all with good clinical outcomes.

**Table 1:** Demographics and summarized baseline clinical data of the study population

Age	Median (range), 7 (1–14), years
Sex	*N* = 6 (55%) males; *N* = 5 (45%) females
Putative etiology of EHPVO	*N* = 8 (73%) UVC *N* = 1 (9%) perinatal sickness without UVC *N* = 2 (18%) unknown
Clinical manifestations of portal hypertension	*N* = 7 (64%) history of gastrointestinal bleeding *N* = 4 (36%) high-risk varices *N* = 11 (100%) hypersplenism
Rex recessus patency	*N* = 4 (36%) yes *N* = 7 (64%) no
Spleno-mesenteric obstruction	*N* = 2 (18%) yes *N* = 9 (82%) no

*EHPVO* extrahepatic portal vein obstruction, *UVC* umbilical vein catheterization

**Table 2:** Procedural data

	*N* = 15 total procedures
Percutaneous approach	*N* = 1 (7%) transhepatic *N* = 11 (73%) transplenic *N* = 3 (20%) simultaneous transhepatic/transplenic
Number of procedures	*N* = 7 (64%) single procedure *N* = 4 (36%) additional procedure
Total fluoroscopy time per procedure	Median (range), 114 (18–178) min
Total DAP per procedure	Median (range), 123 (17–788) Gycm^2^
Overall procedural time	Median (range), 220 (67–358) min
Embolization of varices	*N* = 3 (27%) yes *N* = 8 (73%) no
Technical success	*N* = 5 (45%) yes *N* = 6 (55%) no
Adverse events (according to CIRSE classification system)	*N* = 13 (86%) uneventful procedures *N* = 1 (7%) intrahepatic arterial pseudoaneurysm (grade 3) *N* = 1 (7%) hemoperitoneum (grade 2)

**Table 3:** Outcome data of successful PVR procedures

	*N* = 5 successful PVRs
Rex recessus patency at baseline	*N* = 2
Portal vein patency at last follow-up	*N* = 5
Rex recessus patency at last follow-up	*N* = 3
Follow up time	Median (range), 6 (6–14) months
Clinical manifestations of portal hypertension	*N* = 5 none
Platelet count at baseline	Median (range), 89 (72–174) × 10^9^/L
Platelet count at last follow-up	Median (range), 170 (148–266) × 10^9^/L

## Discussion

Our findings in a preliminary series of PVR in pediatric patients support the consideration of the percutaneous approach for the management of EHPVT. Firstly, PVR can restore the native anatomical condition, while surgical methods rely on bypass creation. Secondly, the Rex recessus patency is necessary for Meso-Rex surgery. In contrast, PVR can be offered regardless of the status of the Rex recessus, given that PVR may restore normal flow through a thrombosed Rex recessus. Thirdly, PVR may achieve clinical resolution of portal hypertension, even when a partial recanalization of the intrahepatic portal system is obtained. Furthermore, percutaneous portal venography provides a dynamic panoramic view of the spleno-mesenteric circulation, allowing the identification of ectopic varices that may be embolized to prevent the flow steal phenomenon. Lastly, it is important to note that a failed percutaneous approach does not interfere with a subsequent surgery, and that Meso-Rex surgery may be accompanied by potential complications, such as obstruction and thrombosis which are eventually managed through percutaneous procedures. Compared to TIPS, PVR offers the advantage of restoring a normal flow to the liver, avoiding the common complications of portosystemic shunts such as over-shunting, hepatic encephalopathy, hepatopulmonary syndrome, and nodule development. With the current knowledge, no disadvantages of PVR, if effective, compared to TIPS may be anticipated.

The decision to proceed with Meso-Rex surgery or PVR is typically multidisciplinary, considering both the clinical picture and imaging findings, with wedge hepatic venography being particularly crucial. Given that interventional radiologists perform this diagnostic examination beforehand, the authors suggest that an attempt at percutaneous PVR might be considered in the same session.

While PVR offers significant advantages, it is essential to note its limitations. At present, no defined criteria exist to predict the technical success of the procedure, which can be straightforward and quick, can last several hours and need additional procedures, or even fail. It has been suggested that the extent of the thrombosed tract and the size of the intrahepatic portal branches may serve as predictors of technical success. Nevertheless, executing these procedures demands advanced technical skills and experience in performing transhepatic, transplenic, or even transmesenteric catheterization of the portal system, currently available only in highly specialized centres. Furthermore, in a significant proportion of technically successful cases, angioplasty may require stenting to maintain the patency of the recanalized portal tract. This, in turn, can pose challenges for a subsequent surgical approach, especially if traditional long stainless-steel stents are employed. However, the availability of new metal alloy devices has partially resolved this concern and the issue of adaptation with child growth. Finally, exposure to ionizing radiation is a relevant concern in pediatric populations, which are more sensitive to potential long-term effects [12], especially for this kind of procedure which often lasts several hours and may require additional interventions. In our series, dosimetry reports highlighted considerable exposure to ionizing radiation, probably also owing to the outdated equipment that has now been replaced. Nevertheless, ionizing radiation exposure is unavoidable during diagnostic and interventional radiological procedures that are often required to manage the complications of Meso-Rex bypass and other surgical shunts. Modern equipment and dedicated pediatric protocols may minimize radiation exposure, which will be the object of investigation in further research.

We acknowledge that the main limitation of this study is the lack of a long-term follow-up, especially if compared to surgical series reporting positive clinical outcomes of Meso-Rex bypass and portosystemic shunt procedures after a median of more than 4 years and 8 years [7, 13]. Nevertheless, literature data about the Meso-Rex bypass feasibility and outcomes is heterogeneous. According to the reported series, the success rate of the Meso-Rex bypass ranges from 60% to 97%, while in the present study, it was judged unfeasible in most cases due to the obstruction or small size of the Rex recessus. Regarding complications, the Meso-Rex surgery may be burdened by a significant rate of thrombosis, which was reported to range between 4% and 13% [6, 7]. Moreover, percutaneous procedures are not uncommonly complicated by access site bleeding, which seems to be rarely reported after surgery. However, a fair comparison of the techniques cannot be done due to the lack of PVR series performed in the same populations of Meso-Rex surgery.

Indeed, a growing body of evidence supports PVR in adults, in native or transplanted livers with positive outcomes in the long-term follow-up [1, 8, 14–19]. Original pediatric series remain scarce [20], but one may hypothesize that most can be extrapolated to the pediatric populations. The available evidence supporting Baveno VII recommendations for managing portal cavernoma in pediatric patients is still limited [5–7, 13] and clinical trials comparing surgery with interventional radiology will offer new evidence to recommend a standard of care.

In view of the very high radiation exposure, the relatively low success rate, and the lack of long-term results, PVR may be considered an option for the time being if a surgical Meso-Rex bypass is not feasible.

Based on the current evidence, local expertise should guide clinical decisions to achieve the best outcomes and interventional radiologists should be aware of innovative percutaneous endovascular procedures that may improve the management of EHPVO and its complications in children not eligible for Meso-Rex bypass surgery.

## Supplementary information

ELECTRONIC SUPPLEMENTARY MATERIAL

## Abbreviations

EHPVO: Extrahepatic portal vein obstruction
PVR: Portal vein recanalization
TIPS: Transjugular intrahepatic portosystemic shunt
