# Supplementary material for: Percutaneous recanalization of non-cirrhotic extrahepatic portal vein obstruction in children: technical considerations in a preliminary cohort
Source: Eur Radiol. 2024 Sep 6;35(6):3262–9. doi: 10.1007/s00330-024-11040-8 (PMC12081556; doi:10.1007/s00330-024-11040-8)
Supplement: Supplementary file 1 — ELECTRONIC SUPPLEMENTARY MATERIAL [file 330_2024_11040_MOESM1_ESM.pdf]

Percutaneous recanalization of non-cirrhotic extrahepatic portal vein obstruction in children: technical considerations in a preliminary cohort

ELECTRONIC SUPPLEMENTARY MATERIAL

Eur Radiol (2024) Marra P, Franchi-Abella S, Hernandez JA, et al.

Table S1. Individual baseline clinical data and procedural data.

ID	Age	Sex	Rex recessus patency	Baseline Endoscopy	History of GI bleeding	Baseline platelet count (x10^3)	Baseline INR	Time between proc. (days)	Proc 1 fluoro time	Proc 1 DAP (Gycm^2)	Proc 1 time	Proc 2 fluoro time	Proc 2 DAP (Gycm^2)	Proc 2 time	Proc 1 access	Proc 2 access	Proc 1 outcome	Proc 2 outcome
1	13	F	no	F2 RC-neg	yes	112	1.5	np	29	140.45	71	np	np	np	Spl	np	failed	np
2	13	F	no	F1 RC-neg	yes	103	1.09	np	135	208.75	228	np	np	np	Spl	np	failed	np
3	14	M	yes	F1 RC-neg	no	72	1.17	64	129	788.14	289	18	112.60	67	Hep + Spl	Spl	PVR + PTA	PTA + stenting
4	7	M	yes	F1 RC-pos	yes	89	1.25	np	115	136.07	209	np	np	np	Spl	np	failed	np
5	5	M	yes	duodenal varices<br>RC-pos	yes	219	1.32	np	126	94.98	358	np	np	np	Hep + Spl	np	failed	np
6	10	F	yes (thin)	F2 RC-neg	yes	81	1.17	np	88	104.08	182	np	np	np	Spl	np	failed	np
7	7	F	yes	F2 RC-pos GV RC-pos	yes	89	1.25	124	124	402.01	313	26	90.93	92	Spl	Spl	PVR + PTA	PTA + stenting
8	4	M	no	F1 RC-neg	yes	80	1.18	np	106	122.69	220	np	np	np	Hep	np	PVR + PTA	np
9	9	M	no	GV RC-pos	yes	129	1.32	5	114	326.30	308	54	236.56	218	Hep + Spl	Spl	PVR + stenting	PVR + PTA
10	1	M	no	F1 RC-pos	yes	174	1.15	21	178	47.02	329	20	17.26	81	Spl	Spl	PVR + PTA	PTA + stenting
11	1	F	yes (thin)	F3 RC-pos	yes	86	1.25	np	166	39.98	284	np	np	np	Spl	np	failed	np

Age expressed in years. Oesophageal and gastric varices graded according to Garcia-Tsao et al [10]. DAP, dose area product; GI, gastrointestinal; GV, gastric varices; Hep, hepatic; INR, international normalized ratio; np, not performed; proc, procedure; PVR, portal vein recanalization; PTA, percutaneous transluminal angioplasty; RC, red colour sign; Spl, splenic.

Table S2. Individual technical details and clinical outcomes data.

ID	Tech success	Final manag.	Catheter microcath. (French)	Guide-wire	PTA balloon extrahepatic	PTA balloon intrahepatic	PV stent	Variceal emboliz.	Complicat-ions/ Manag.	Anticoagul-ation therapy	Imaging follow-up (months)	PV paten-cy	Last platelet count	Last Endoscopy	Gl ble at up
1	no	Splenect-omy	Straight 2.7F	Hydroph. 0.035"	np	np	np	yes	none	Prophylactic LMWH (3 months)	4	no	439	np	no
2	no	TIPS	Straight 1.9F	Hydroph. 0.014"-0.018"	np	np	np	yes	none	no	12	no	144	Eradicated	no
3	yes	PVR	Straight 1.9F	Hydroph. 0.014"-0.018"	NC 12 mm x 6 cm	np	Wallstent 12 mm x 4 cm	yes	none	Antiplatelet (continued)	14	yes*	162	F0 RC-neg	no
4	no	Follow up	Straight 1.9F	Hydroph. 0.014"-0.018"	np	np	np	no	none	no	12	no	80	F1 RC-pos GOV1	no
5	no	TIPS	Straight 1.9F	Hydroph. 0.014"-0.018"	np	np	np	no	none	Prophylactic LMWH (3 months)	10	no	113	Eradicated	no
6	no	Follow up	Straight 1.9F	Hydroph. 0.014"-0.018"	np	np	np	no	none	no	0	no	63	np	no
7	yes	PVR	Straight 1.9F	Hydroph. 0.014"-0.018"	NC 10 mm x 6 cm	NC 8 mm x 6 cm	Omnilink Elite 10 mm x 29 mm	no	HA PA/ Emboliz.	LMWH (6 months)	10	yes*	204	np	no

**8**	**yes**	**PVR**	**Straight 1.9F**	**Hydroph. 0.014"-0.018"**	**NC 10 mm x 6 cm**	**NC 6 mm x 6 cm**	**np**	**yes**	**none**	**LMWH (continued)**	**6**	**yes**	**266**	**Eradicated**	**no**
**9**	**yes**	**PVR**	**Straight 1.9F Cobra 1.8F**	**Hydroph. 0.014"-0.018"**	**NC 10 mm x 6 cm**	**NC 8 mm x 6 cm**	**Omnilink Elite 10 mm x 29 mm**	**yes**	**none**	**LMWH (3 months)**	**6**	**yes***	**148**	**np**	**no**
**10**	**yes**	**PVR**	**Straight 1.9F**	**Hydroph. 0.014"-0.018"**	**NC 8 mm x 4 cm**	**NC 5 mm x 4 cm**	**Omnilink Elite 8 mm x 19 mm**	**no**	**Hemoperit-oneoum/ conserv.**	**LMWH (3 months)**	**6**	**yes***	**170**	**np**	**no**
**11**	**no**	**MRS**	**Straight 1.9F**	**Hydroph. 0.014"-0.018"**	**np**	**np**	**np**	**no**	**none**	**LMWH (continued)**	**1**	**no**	**154**	**np**	**no**

Oesophageal and gastric varices graded according to Garcia-Tsao et al. [10]. *primary-assisted patency. GI, gastrointestinal; GOV, gastroesophageal varices; LMWH, low molecular weight heparin; MRS, surgical mesorenal shunt; NC, non-compliant; np, not performed; PA, pseudoaneurysm; proc, procedure; PV, portal vein; PVR, portal vein recanalization; PTA, percutaneous transluminal angioplasty; RC, red colour sign; TIPS, transjugular intrahepatic portosystemic shunt.
